# RNA-sequencing approach for exploring the protective mechanisms of dexmedetomidine on pancreatic injury in severe acute pancreatitis

**DOI:** 10.3389/fphar.2023.1189486

**Published:** 2023-05-11

**Authors:** Jiaqi Yao, Bowen Lan, Chi Ma, Yan Liu, Xiaoqi Wu, Kaixuan Feng, Hailong Chen, Qingping Wen

**Affiliations:** ^1^ Department of Anesthesiology, The First Affiliated Hospital of Dalian Medical University, Dalian, Liaoning, China; ^2^ Laboratory of Integrative Medicine, The First Affiliated Hospital of Dalian Medical University, Dalian, Liaoning, China; ^3^ Department of General Surgery, The First Affiliated Hospital of Dalian Medical University, Dalian, Liaoning, China; ^4^ Department of Anesthesiology, Affiliated Xinhua Hospital of Dalian University, Dalian, China; ^5^ College (Institute) of Integrative Medicine, Dalian Medical University, Dalian, Liaoning, China

**Keywords:** severe acute pancreatitis, dexmedetomidine, inflammation, RNA sequencing, mechanisms

## Abstract

**Background:** Severe acute pancreatitis (SAP) is a severe form of acute pancreatitis with the potential to cause life-threatening complications. Patients with acute SAP require surgical intervention and are admitted to the intensive care unit for non-invasive ventilation. Dexmedetomidine (Dex) is currently used by intensive care clinicians and anaesthesiologists as an adjunctive sedative. Therefore, the clinical availability of Dex makes it easier to implement in SAP treatment than developing new drugs.

**Methods:** Randomly dividing thirty rats into sham-operated (Sham), SAP, and Dex groups. The severity of pancreatic tissue injury in each rat was assessed by Hematoxylin and eosin (HE) staining. Serum amylase activity and inflammatory factor levels were measured using commercially available kits. The expressions of necroptosis-related proteins, myeloperoxidase (MPO), CD68, and 4-hydroxy-trans-2-nonenal (HNE) were detected using immunohistochemistry (IHC). Transferase-mediated dUTP nick-end labeling (TUNEL) staining was utilized to identify pancreatic acinar cell apoptosis. The subcellular organelle structure of pancreatic acinar cells was observed using transmission electron microscopy. The regulatory effect of Dex on the gene expression profile of SAP rat pancreas tissue was investigated using RNA sequencing. We screened for differentially expressed genes (DEGs). Quantitative real-time PCR (qRT-PCR) measured critical DEG mRNA expression in rat pancreatic tissues.

**Results:** Dex attenuated SAP-induced pancreatic injury, infiltration of neutrophils and macrophages, and oxidative stress. Dex inhibited the expression of necroptosis-associated proteins RIPK1, RIPK3, and MLKL and alleviated apoptosis in acinar cells. Dex also mitigated the structural damage caused by SAP to mitochondria and endoplasmic reticulum. Dex inhibited SAP-induced 473 DEGs, as determined by RNA sequencing. Dex may regulate SAP-induced inflammatory response and tissue damage by inhibiting the toll-like receptor/nuclear factor κB (TLR/NF-κB) signaling pathway and neutrophil extracellular trap formation.

**Conclusion:** This study elucidated the remarkable effect of Dex against SAP and investigated the potential mechanism of action, providing an experimental base for the future clinical application of Dex in the treatment of SAP.

## 1 Introduction

Acute pancreatitis (AP) is a common digestive disease with an increasing trend in incidence. A total of 330,000 cases of AP are reported annually in the United States (Horibe, et al., 2022). Severe acute pancreatitis (SAP) is a severe form of AP that may result in life-threatening complications. The prognosis for individuals with SAP is poor due to the severe symptoms, systemic inflammatory responses, and multi-organ failure. SAP is mainly treated with symptomatic therapy and prevention of complications (Ge, et al., 2020). Nevertheless, hospitalization and access to advanced medical technology are expensive and time-consuming, interrupting patients’ lives and imposing a substantial weight on health insurance (Gui, et al., 2023). Early diagnosis of SAP and early medical intervention may significantly improve the patient’s prognosis and avoid complications. Therefore, it is advantageous to develop novel SAP treatments.

Dexmedetomidine (Dex) is often used to treat critical care, sedation, pain, anxiety, and insomnia due to its ability as an alpha2-adrenergic receptor agonist (Baumgartner, et al., 2022; Moore, et al., 2022; Xiao, et al., 2023). Numerous organs, including the brain, heart, lungs, kidney, liver, and small intestine, are protected by Dex (Xia, et al., 2017; Jiang, et al., 2019; Liu, et al., 2019). Dex may become a potential therapeutic multi-organ protection medication based on its extensive clinical use and safety (Liu, et al., 2021). Dex protects cells greatly against several stresses by activating the anti-apoptotic signal pathway and considerably regulating systemic inflammatory response syndrome by decreasing the release of pro-inflammatory cytokine. In addition, Dex has been discovered to perform immunomodulatory effects by inhibiting activated immune cells such as T cells and macrophages (Chen, et al., 2022). Dexmedetomidine pretreatment exerts protective efects on rat pancreas irradiated abdominally with ionizing radiation (Mercantepe, et al., 2023). Multiple preclinical investigations have shown that Dex has a remarkable therapeutic effect in animal models of AP. Li, et al. (2018) discovered that by suppressing the assembly of NOD-like receptor protein 3 (NLRP3) inflammasome, Dex might mitigate pancreatic damage produced by the combination injection of cerulein and lipopolysaccharide (LPS). In addition, Huang, et al. (2020) found that via modulating the cholinergic anti-inflammatory system, Dex may attenuate the inflammatory response of SAP rats induced by sodium taurocholate (STC). Dex is a potential treatment for SAP. Clarifying the molecular mechanism by which Dex ameliorates local pancreatic damage and the systemic inflammatory response of SAP might give a theoretical foundation for the drug’s novel use. In this study, the anti-inflammatory and organ-protective benefits of Dex were initially evaluated in SAP rats. Second, we utilized transcriptomics and molecular biology to figure out the effect of Dex on necroptosis during SAP and the molecular pathways underlying this action.

## 2 Materials and methods

### 2.1 Reagent and instrument

Dexmedetomidine was obtained from Hengrui Medicine. Interleukin-1 beta (IL-1β), interleukin-6 (IL-6), and tumor necrosis factor-α (TNF-α) Enzyme-linked immunosorbent assay (ELISA) kits were obtained from Xitang Co. Ltd. Serum amylase assay kits were obtained from Nanjing Jiancheng. Anti-RIPK3 antibodies were obtained from ABclonal. Anti-RIPK1, MLKL, GAPDH, MPO, CD68, 4-HNE, and sheep anti-rabbit IgG (H + L) HRP were obtained from Sanying Co. Ltd. Hematoxylin and eosin (HE) staining kit was obtained from Beyotime. Fluorescence quantitative PCR instrument was obtained from Applied Biosystems.

### 2.2 Animals and groups

The rats were raised in ambient conditions and had *ad libitum* access to standard laboratory pelleted formula and tap water. All rats were placed in a room (12/12 h light-dark cycle, 21°C ± 2°C, humidity 50% ± 10%) and adaptively fed for 1 week before the experiment. All the animal experiments conformed with the Guidelines for the Care and Use of Laboratory Animals issued by the National Academy of Sciences, published by the National Institute of Health. The experimental protocol was approved by the Ethics Committee for Animal Experimentation of Dalian Medical University. The ethical approval reference number of the study is 110322210101357521. The Sprague-Dawley rats in this study were acquired from the Huafukang Co. Randomly; thirty rats were divided into three groups: sham-operated (Sham), SAP, and Dex. The random number sequence was generated using a table of random numbers. The SAP and Dex groups received retrograde injections of 5% sodium taurocholate (STC) into the biliopancreatic duct. The control animals (sham group) were treated with the same volume of normal saline (0.9% NaCl). After 2 h, the rats in the Dex group were injected intraperitoneally with Dex (10 μg/kg). All rats were sacrificed 24 h after the first treatment, and blood samples and pancreatic tissue were obtained for further testing.

### 2.3 Histopathological assessment

The pancreatic tissue was fixed overnight at room temperature with formalin, then embedded in paraffin and sliced using a tissue microtome. Next, tissue sections (4 µm) were used to perform an HE assay. As previously described (Schmidt, J et al., 1992), pancreatic tissue sections were scored for the severity of pancreatitis based on edema (0–4 points), leukocyte infiltration, acinar cell necrosis (0–4 points), and hemorrhage (0–4 points). The individual scores were then added to obtain the total pathological score for the pancreatic tissue.

### 2.4 Detection of amylase activity and inflammatory factors

The blood samples were centrifuged at 1,000 *g* for 15 min at 4°C to collect serum. The levels of serum amylase and inflammatory factors were measured using commercial kits.

### 2.5 Immunohistochemistry assay

The pancreatic tissue sections (4 μM) were dewaxed twice with xylene and dehydrated for 5 min with gradient alcohol. After three washes with double steaming water, antigen retrieval with citrate sodium is performed. The tissue slices are then blocked with a blocking buffer for 30 min at room temperature. Incubate primary and secondary antibodies separately. Hematoxylin was employed to stain the nucleus after the primary antibody-labeled protein was stained with 3,3′-diaminobenzidine (DAB). An inverted fluorescent microscope was used to get the pictures.

### 2.5 Transmission electron microscopy

The pancreas tissues were fixed in 2.5% glutaraldehyde for 2 h, followed by overnight fixation at 4°C. After dehydration, embedding, and solidification, the ultrastructural structure was observed using a Hitachi HT7800 transmission electron microscope (Japan).

### 2.6 TUNEL

Prepared pancreatic slides (4 M) were dewaxed with xylene twice, washed with phosphate-buffered saline solution (PBS) twice, then dehydrated with gradient alcohol (5 min). Subsequently, 0.3% Trixon-X100 was used to penetrate cells at room temperature (5 min). Following two washes with PBS, apoptotic cells were stained with TUNEL buffer (Roche Diagnostics GmbH, Roche, Germany) for 1 h at 37°C. The nuclei were stained using Antifade Mounting Medium with 4,6-diamidino-2-phenylindole (DAPI) (Sigma, United Kingdom), and the cells were mounted. The photos were captured using an inverted fluorescent microscope.

### 2.7 RNA sequencing

RNA sequencing (RNA-seq) on pancreatic tissue from rats in the Sham, SAP, and Dex groups that had been digested into single cells and resuspended in 1 mL TRIzol reagent (Invitrogen, Carlsbad, CA). Sending RNA samples for library creation and RNA sequencing, the Illumina HiSeq 2000 platform was used to produce expression libraries with a 150-nt read length. To prevent random mistakes, we had three separate RNA sequencing samples.

### 2.8 Identification and analysis of DEGs

Genes with |log_2_ fold change|>1 and *p* < 0.05 were selected as DEGs and shown as volcano plots. Venn diagrams of DEGs for SAP vs. Sham and Dex vs. SAP were plotted to obtain intersecting DEGs. DEGs that overlapped were uploaded into the DAVID database for Gene Ontology (GO) functional analysis and Kyoto Encyclopedia of Genes and Genomes (KEGG) pathway analysis, and histograms were plotted. The overlap DEGs were further uploaded into the STRING database. Construct a protein-protein interaction (PPI) network using Cystocape 8.2 software. Gene cluster modules with K-score greater than three were screened using the MCODE plugin. The data from the SAP and Dex groups were analyzed using the GSEA 4.2.3 program for Gene Set Enrichment Analysis (GSEA) with a random combination set to 100 times and screening conditions of *p* < 0.05 and |NES|>1 to obtain significantly enriched signaling pathways.

### 2.9 Quantitative RT-PCR assay

RNA TRIzol was used to extract total RNA from each group of rats’ pancreas and then for further analysis. Reverse transcription was performed according to PrimeScript™ RT reagent Kit (TAKARA), and qRT-PCR assay was employed in the SYBR green kit (TAKARA) at Bio-Rad CFX96 PCR System. The primer sequences of all genes are shown in Table 1. GraphPad Prism 8 was used for qRT-PCR data analysis.

**TABLE 1:** The primer sequences of all genes.

	Forward	Reverse
*aqp9*	CTC​GGA​CTC​AAC​TCT​GGC​TG	GAC​CCA​CGA​CAG​GTA​TCC​AC
*cxcl1*	GGT​GAG​GAA​ATG​TGT​GAG​AGG	AGA​CGA​GAA​GGA​GCA​TTG​GTT
*cxcr2*	ACA​GAG​ACT​TGG​GAG​CCA​CT	GAC​AGA​GTA​AAG​GGC​GGG​TC
*IL-6*	ATT​GTA​TGA​ACA​GCG​ATG​ATG​CAC	CCA​GGT​AGA​AAC​GGA​ACT​CCA​GA
*IL-1β*	GGC​AAC​TGT​CCC​TGA​ACT​CAA	GCT​TCT​CCA​CAG​CCA​CAA​TGA
*s100a8*	GGG​AAT​CAC​CAT​GCC​CTC​TAC	TCT​TTA​TGA​GCT​GCC​ACG​CC
*s100a9*	CCT​GAC​ACC​CTG​AAC​AAG​GC	TCC​CAT​CAG​CAT​CAT​ACA​CTC​C
*trem1*	TGA​CCA​AGG​GTT​CTT​CAG​GTG	GTG​GAG​ACA​CTC​GTA​GGA​TCT​G
*cldn5*	CAT​CGG​TGA​AGT​AGC​CAC​CA	TGC​CCT​TTC​AGG​TTA​GCA​GG
*gapdh*	CTG​GAG​AAA​CCT​GCC​AAG​TAT​G	GGT​GGA​AGA​ATG​GGA​GTT​GCT

### 2.10 Statistical analysis

All experimental data in the present investigation were collected independently three times and presented as the mean standard deviation (SD). GraphPad Prism 8 was used to analyze all experimental data in this work. The statistical analysis of the quantitative multigroup was compared using the one-way ANOVA. Differences were considered statistically significant at *p* < 0.05 or *p* < 0.01.

## 3 Results

### 3.1 Dexmedetomidine inhibits pancreatic injury and inflammation in SAP rats

**FIGURE 1:**
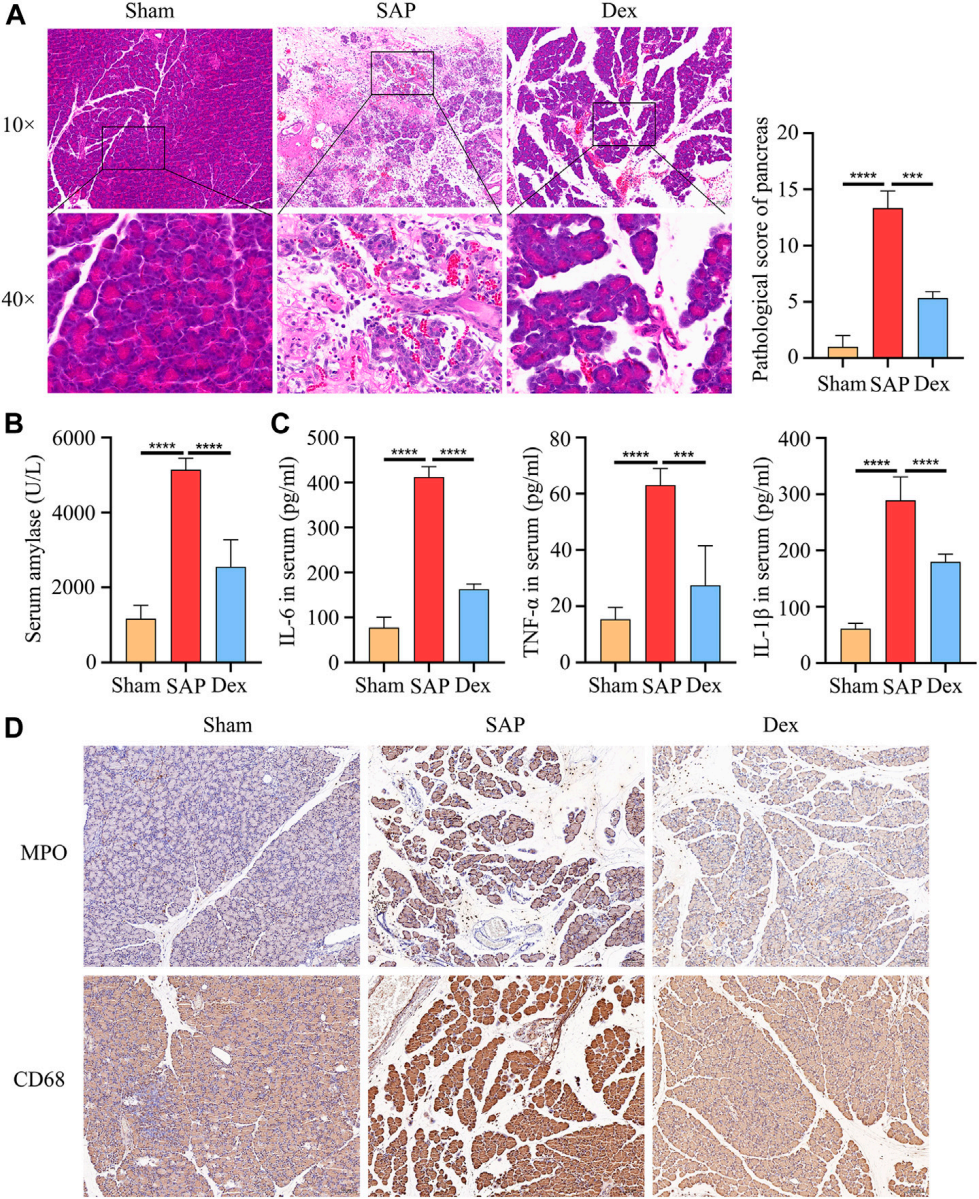
Dexmedetomidine inhibits pancreatic injury and inflammation in rats. **(A)** HE staining shows the tissue structure of SAP rats treated with dexmedetomidine. **(B)** The amylase activity of serum in each group of rats. **(C)** ELISA assay shows the inflammation factor of serum in each group of rats. **(D)** IHC staining to detect the MPO and CD68 protein expression in each group of rats. Data are presented as means ± SD (*n* = 3). ***p < 0.001, ****p < 0.0001.

Rats with severe acute pancreatitis are often infiltrated with many inflammatory cells. In contrast, Dex significantly improved pancreatic tissue’s bleeding, necrosis, and inflammatory cell infiltration. Significantly lower pancreatic pathological damage scores in the Dex group (Figure 1A). Amylase is one of the gold criteria for diagnosing SAP. Serum amylase levels were increased more than 3-fold in our established SAP rat model compared to the Sham group. Dex significantly decreased serum amylase activity (Figure 1B). Similarly, serum inflammatory factors reflect the systemic inflammatory response of SAP rats. We tested the serum IL-1β, IL-6, and TNF-α levels in each group of rats. Dex reduced the upregulation of inflammatory factors induced by SAP (Figure 1C). Myeloperoxidase (MPO) is a marker of neutrophil infiltration. CD68 is a marker of macrophage activation. Dex significantly inhibited the excessive activation of neutrophils and macrophages by SAP (Figure 1D).

### 3.2 Effect of Dex on oxidative stress, apoptosis and subcellular organelle structure of rat pancreatic acinar cells

**FIGURE 2:**
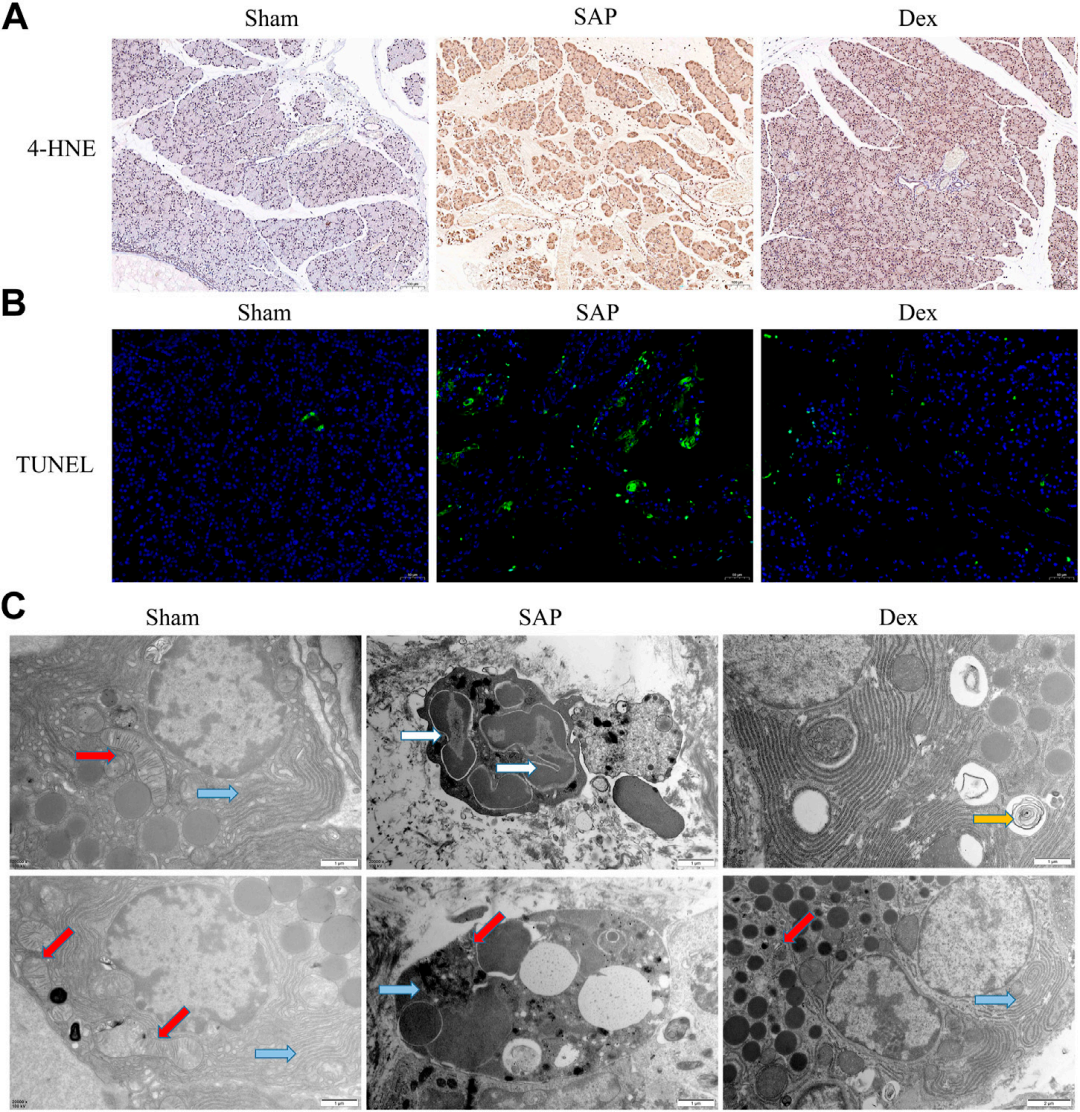
Effect of Dex on oxidative stress, apoptosis and subcellular organelle structure of rat pancreatic acinar cells. **(A)** IHC staining was used to detect the expression of 4-HNE, one marker of oxidative stress, in each group of rats. **(B)** TUNEL assay shows the apoptosis cell number in each group of rats. **(C)** Transmission electron microscope observes the structure of mitochondria and endoplasmic reticulum in each group of rats. Blue arrows, endoplasmic reticulum; red arrows, mitochondria; white arrows, nuclear fragmentation; yellow arrow, autophagosome.

Mitochondria and endoplasmic reticulum are energy supply centers and protein processing plants and mediate various biological processes, such as regulating calcium homeostasis, autophagy, and oxidative stress. Under physiological conditions, appropriate oxidative stress is necessary to maintain normal cellular function. When cellular mitochondrial and endoplasmic reticulum structures are damaged, sustained and excessive oxidative stress, large amounts of reactive oxygen species can damage cellular proteins and DNA and cause cell death. 4-Hydroxynonenal (4-HNE) is a standard indicator to assess oxidative stress. Our results showed that 4-HNE expression was elevated in the pancreatic tissue of rats in the SAP group. Dex administration downregulated 4-HNE expression (Figure 2A). Acinar cell apoptosis is a key player in the development of SAP. We used TUNEL staining to observe apoptosis in pancreatic tissue. The results showed that Dex significantly inhibited SAP-induced apoptosis in pancreatic acinar cells (Figure 2B). We also observed the subcellular organelle structure of pancreatic acinar cells using transmission electron microscopy. The ultrastructure of rat pancreatic tissue was normal in the Sham group, while the SAP group showed neutrophil nuclear division, apoptotic bodies in the nucleus, cytoplasmic vacuole degeneration, mitochondrial swelling and endoplasmic reticulum expansion. In the Dex group, the pathological changes of acinar cells were alleviated, the mitochondria were slightly swollen, and the structural damage of the endoplasmic reticulum was also improved (Figure 2C).

### 3.3 Inhibitory effects of dex on necroptosis in SAP rats

**FIGURE 3:**
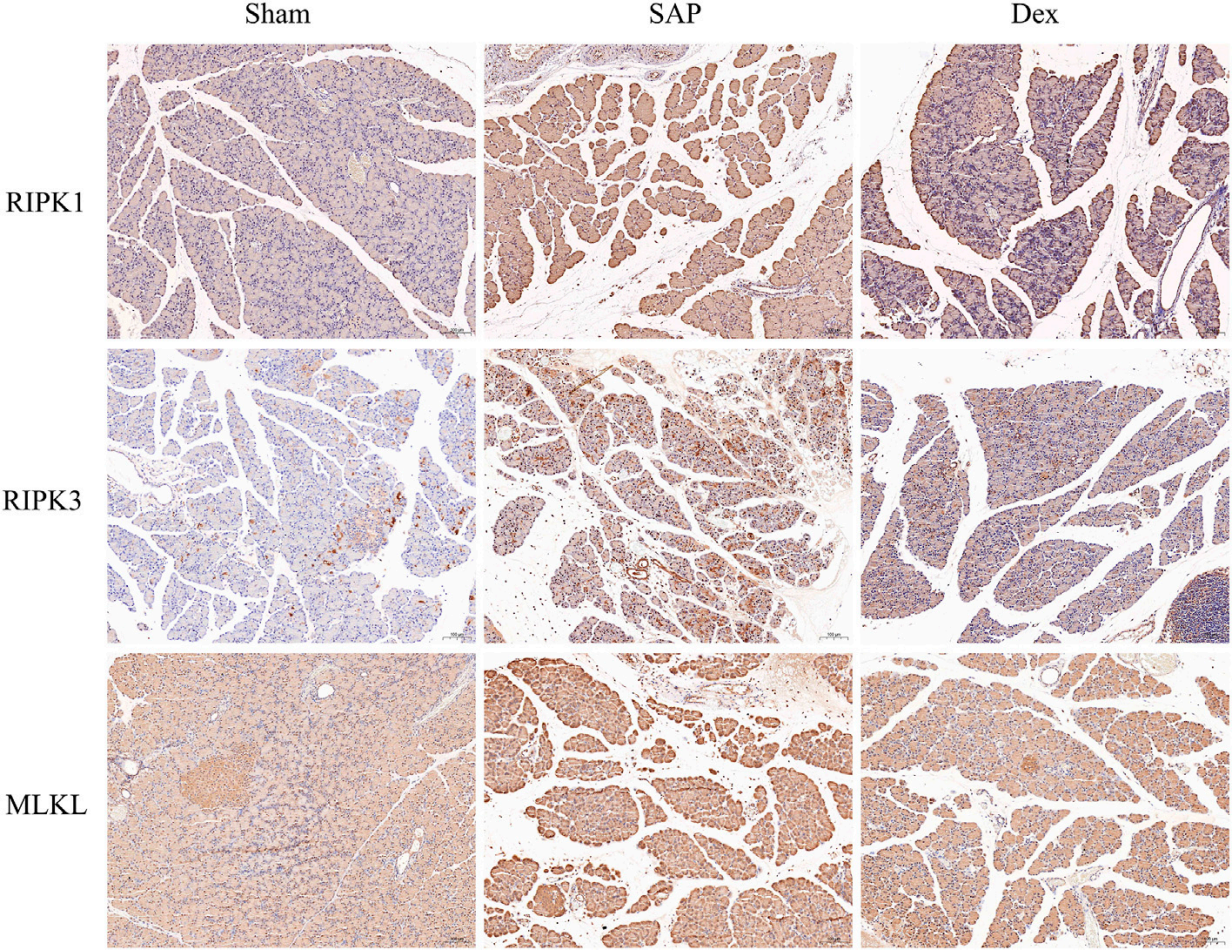
Inhibitory effects of Dex on necroptosis in SAP rats. Immunohistochemistry assay detects the necroptosis-related protein expression in each group of rats.

Necroptosis is intimately related to the development of several diseases, especially cardiovascular, digestive, neurological, and respiratory conditions (Leak and Dixon, 2023). Necroptosis in pancreatic acinar cells is a crucial factor in initiating AP. Targeting necroptosis is a possible therapeutic option for SAP (He, et al., 2022). We used immunohistochemistry to detect protein markers of necroptosis, including receptor-interacting protein kinase 1 (RIPK1), receptor-interacting protein kinase 3 (RIPK3), and mixed lineage kinase-like domain (MLKL) in the pancreatic tissues. The protein expression was increased in pancreatic tissues of the SAP group. Dex significantly downregulated the expression of the above three marker proteins. (Figure 3).

### 3.4 Effect of Dex on DEGs in pancreatic tissue of SAP rats

**FIGURE 4:**
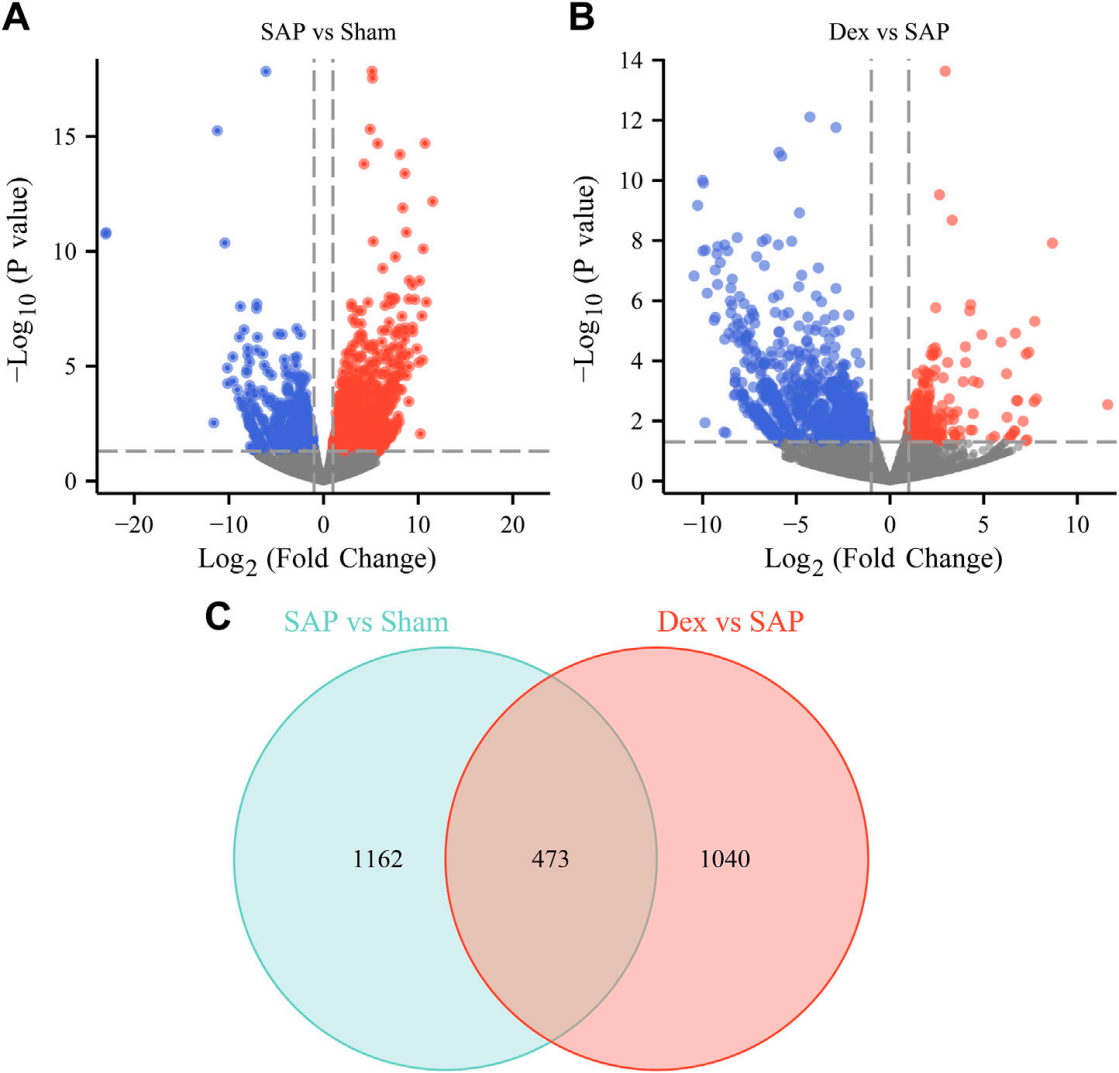
Effect of Dex on DEGs in pancreatic tissue of SAP rats. **(A)** The volcano plot shows the DEGs between the Sham and SAP groups. **(B)** The volcano plot shows the DEGs between the Dex and SAP groups. **(C)** Venn diagram shows overlapping DEGs between Sham vs. SAP DEGs and Dex vs. SAP DEGs.

RNA sequencing was performed to investigate the potential regulatory mechanisms of Dex’s effect on the crosstalk between inflammatory response, necroptosis, and oxidative stress in anti-SAP. The RNA-sequencing results showed 1635 DEGs (1037 upregulated and 598 downregulated) in the SAP group (Figure 4A). There were 1513 DEGs (309 upregulated and 1204 downregulated) in the Dex group (Figure 4B). The DEGs among the three groups were subjected to intersection analysis. It was found that Dex regulated SAP-associated 473 DEGs (Figure 4C).

### 3.5 Validation of critical DEGs

**FIGURE 5:**
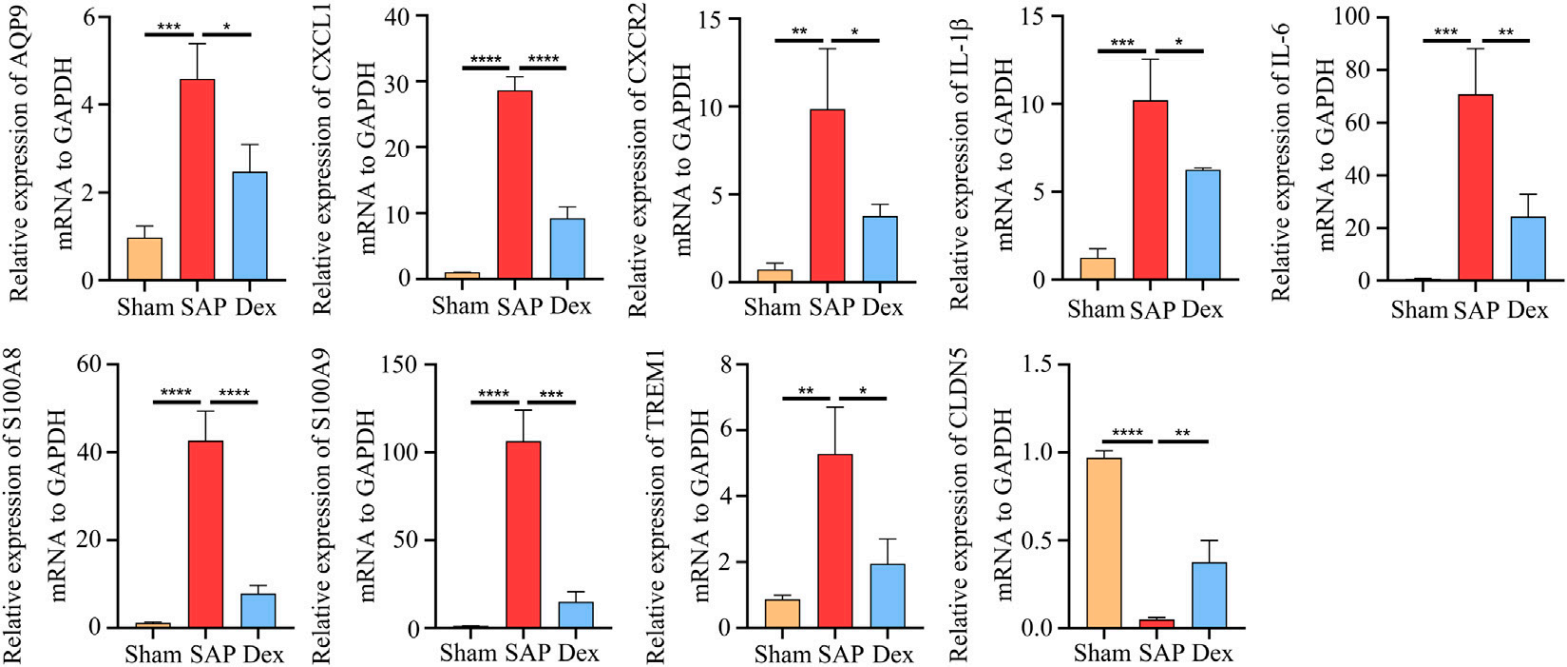
Validation of critical DEGs. Statistical charts show the mRNA expression of critical DEGs in Sham, SAP, and Dex groups. Data are presented as means ± SD (*n* = 3). ^*^ *p* < 0.05, ^**^ *p* < 0.01, ^***^ *p* < 0.001, ^****^ *p* < 0.0001.

To further prove the results of our analysis, we screened the most critical overlapping DEGs between the Dex and SAP groups and then used qRT-PCR to detect their expression in the pancreatic tissues. In the SAP group, the expression of aquaporin 9 (AQP9), C-X-C motif chemokine ligand 1 (CXCL1), C-X-C motif chemokine receptor 2 (CXCR2), IL1B, IL6, S100 calcium-binding protein A8 (S100A8), S100 calcium-binding protein A9 (S100A9), and triggering receptor expressed on myeloid cells-1 (TREM-1) was increased, whereas it was decreased in the Dex group. In contrast to the SAP group, tight junctional protein claudin 5 (CLDN5) expression was raised in the Dex group (Figure 5).

### 3.6 Enrichment analysis and clustering analysis of DEGs

**FIGURE 6:**
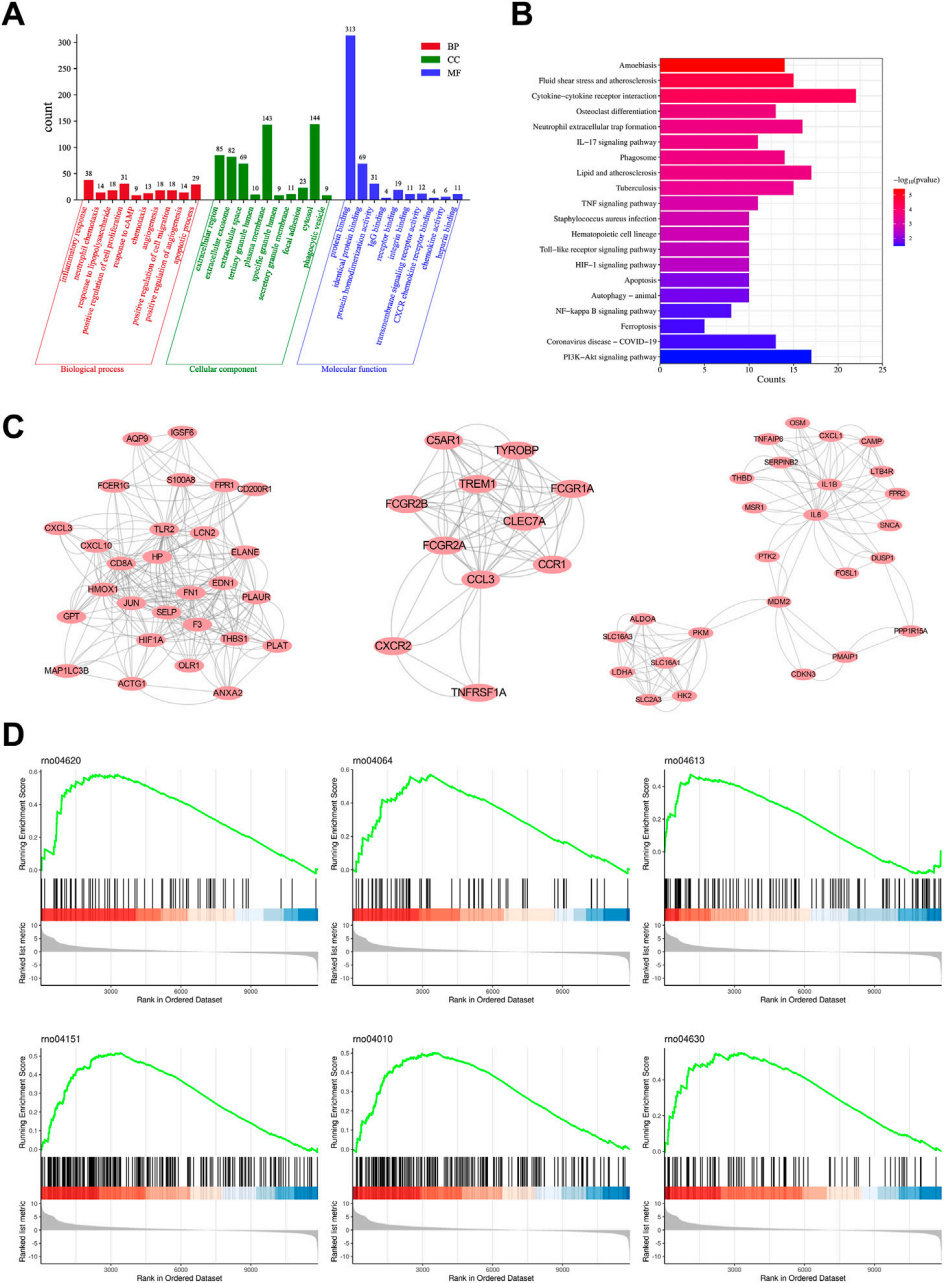
Enrichment analysis and clustering analysis of DEGs. **(A)** GO analysis shows the primary biological process, molecular function, and cellular component in overlapping DEGs. **(B)** KEGG pathway analysis shows the main pathway in overlapping DEGs. **(C)** Three gene modules with K-score greater than 3 of overlapping DEGs. **(D)** GSEA analysis shows the enriched pathway in Dex and SAP groups.

**FIGURE 7:**
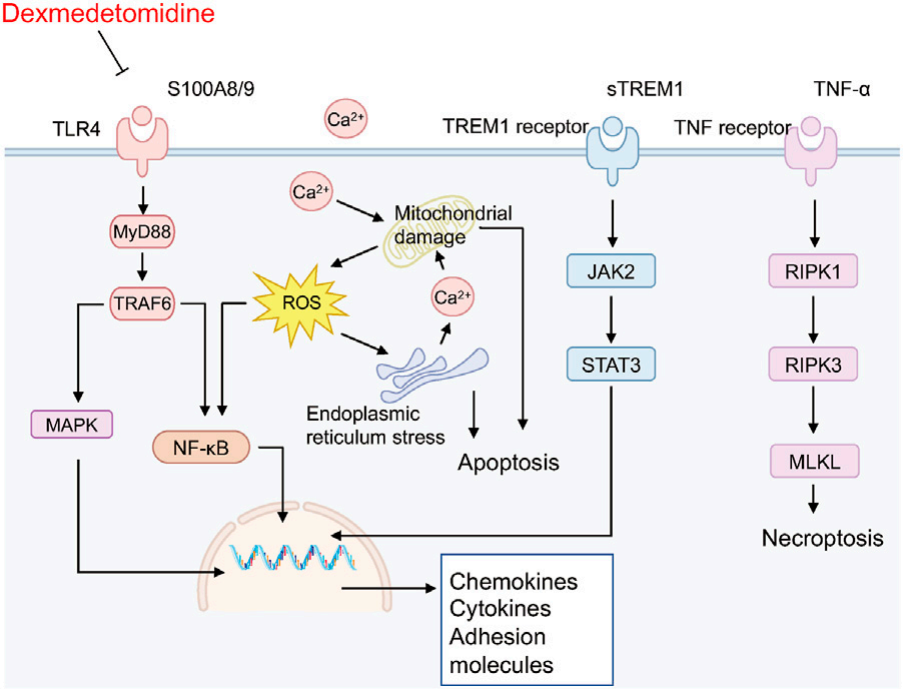
Potential mechanism of dexmedetomidine inhibiting inflammation and pancreatic injury in SAP rats.

To further identify the main targets and possible pathways of Dex treatment in SAP rats, the DEGs between the three groups were taken for intersection analysis. The results showed that Dex significantly regulated SAP-associated 473 DEGs (Figure 4A). Next, we performed GO and KEGG enrichment analysis of DGEs using the DAVID database. DEGs were enriched in biological processes such as inflammatory response, neutrophil chemotaxis, and response to lipopolysaccharide (Figure 6A). DEGs were enriched in the neutrophil extracellular trap (NET) formation, TLR, and NF-κB signaling pathway (Figure 6B). We further performed gene clustering analysis of DEGs using the MOCDE plugin and identified three gene modules with K-score greater than 3, with scores of 9.185, 7.4, and 4.96, respectively (Figure 6C). GSAE analysis showed that Dex mainly regulates NET formation, TLR, NF-κB, Janus kinase-signal transducer and activator of transcription pathway (JAK/STAT), phosphoinositide 3-kinase/protein kinase B (PI3K/AKT), and mitogen-activated protein kinase (MAPK) signaling pathway (Figure 6D). Potential mechanism of dexmedetomidine inhibiting inflammation and pancreatic injury in SAP rats was summarized in Figure 7.

## 4 Discussion

SAP is a severe, potentially life-threatening form of acute abdomen characterized by the potential involvement of other organs and systems. Early detection and active treatment are essential to improve the prognosis of severe acute pancreatitis. In the acute phase, SAP patients usually require admission to the intensive care unit for symptomatic treatment such as mechanical ventilation, fluid resuscitation, or renal replacement therapy (Yang, et al., 2023). Dex reacts with α2 adrenergic receptors in the central nervous system, decreases sympathetic activity, leading to a shift in balance to the parasympathetic nervous system (including the vagus nerve), and is widely used in the intensive care unit (Wiegand, et al., 2023). This study elucidated the remarkable effect of Dex against SAP and investigated the potential mechanism of action by RNA sequencing.

We constructed a rat model of SAP using 5% STC retrogradely injected into the biliopancreatic duct. The results of HE staining, pathological scoring, serum amylase, and inflammatory factor assays showed that the SAP model was successfully replicated. In contrast, Dex significantly alleviated pain, reduced pathological pancreatic damage, and decreased serum amylase activity and inflammatory factor levels in SAP rats. In addition, transmission electron microscopy results showed that the pancreatic tissue of SAP rats appeared neutrophil nuclear division, apoptotic bodies in the nucleus, cytoplasmic vacuole degeneration, mitochondrial swelling, and endoplasmic reticulum expansion. In SAP, oxidative stress is considered as an essential factor in the development of the disease (Liu, et al., 2022). Antioxidant therapy, such as the administration of antioxidants or precursors of the antioxidant system, has been recommended as a potential strategy to alleviate SAP (Zheng, et al., 2022). Here, our findings suggest that Dex may inhibit SAP-induced oxidative stress by ameliorating structural damage to subcellular organelles in pancreatic acinar cells. Necroptosis has been considered as pro-inflammatory cell death and is responsible for tissue damage and the development of chronic inflammation (Tovey Crutchfield, et al., 2021). Here, we discovered Dex inhibited the SAP inflammation process by influencing necroptosis.

Transcriptomic sequencing technology provides a new direction for exploring therapeutic drug targets. In this work, we discovered the targets and mechanisms of Dex action by mining the regulatory effects of Dex on the mRNA expression profile of pancreas tissue. We used RNA sequencing for the first time to study the regulatory effect of dexmedetomidine on the gene expression profile of pancreatic tissue in SAP rats. Specifically, Dex significantly regulated 473 SAP-associated DEGs. The expression of S100 calcium-binding protein A8 (S100A8) and S100A9 was significantly elevated in the peripheral venous blood of patients with AP and had potential diagnostic value (Nesvaderani, et al., 2022). Xiang, et al. (2021) found that S100A9 may amplify the inflammatory response by promoting NLRP3 inflammasome assembly to induce pancreatic ductal cell injury. In addition, it was reported that exosome-specific S100A8/9 might be a key molecule mediating oxidative stress and inflammatory response in AP (Carrascal, et al., 2022). The triggering receptor expressed on myeloid cell 1 (TREM1) is a membrane receptor associated with inflammation and infection. It has been reported that Soluble TREM-1 contributes to the rapid and accurate diagnosis of necrotic tissue secondary infection in patients with SAP (Liu, et al., 2015). TREM-1 is also a key molecule mediating AP-associated intestinal barrier damage (Dang, et al., 2012). We examined the expression levels of DEGs including AQP9, CXCL1, CXCR2, IL1B, IL6, S100A8, S100A9, TREM1, and CLDN5 in pancreatic tissues using qRT-PCR. The results were consistent with RNA seq.

Mechanistically, DEGs were significantly enriched in biological processes such as inflammatory response, neutrophil chemotaxis, and response to lipopolysaccharide. In addition, DEGs were enriched considerably in NET formation, TLR, and NF-κB signaling pathways. We further identified the signaling pathways regulated during Dex treatment of SAP using GSEA analysis. The results were similar to the KEGG enrichment analysis, and the signaling pathways included the Toll-like receptor signaling pathway, NF-κB signaling pathway, NET formation, PI3K-Akt signaling pathway, and MAPK signaling pathway. Toll-like receptor-mediated activation of the NF-κB pathway is the backbone signal that induces the inflammatory cascade response in SAP (Ferrero-Andrés, et al., 2020; Wang, et al., 2022). Numerous studies have reported the potential of inhibiting TLRs/NF-κB signaling pathways in the treatment of SAP. NETs are reticulated ultrastructures released by neutrophils to fight pathogen infection. Accumulating evidence suggests that the massive release of NETs is responsible for organ damage in SAP (Li, et al., 2022). Targeting NETs is a potential strategy for the treatment of SAP (Kang, et al., 2022; Hu, et al., 2020).

## 5 Conclusion

This study elucidated the remarkable effect of Dex against SAP. We used RNA sequencing for the first time to study the regulatory effect of dexmedetomidine on the gene expression profile of pancreatic tissue in SAP rats, and providing an experimental base for the future clinical application of Dex in the treatment of SAP.

However, there are many shortcomings in our work, including but not limited to 1) the lack of *in vitro* experiments to verify the protective effect of Dex on necroptosis injury; 2) the mechanism of Dex on TLRs/NF-κB signaling pathway and NETs release needs to be further confirmed.

## Data Availability

The original contributions presented in the study are included in the article/Supplementary Material further inquiries can be directed to the corresponding authors.

## References

[B1] Baumgartner K. Groff V. Yaeger L. H. Fuller B. M. (2022). The use of dexmedetomidine in the emergency department: A systematic review. Acad. Emerg. Med. 30, 196–208. 10.1111/acem.14636 36448276

[B2] Carrascal M. Areny-Balagueró A. de-Madaria E. Cárdenas-Jaén K. García-Rayado G. Rivera R. (2022). Inflammatory capacity of exosomes released in the early stages of acute pancreatitis predicts the severity of the disease. J. pathology 256, 83–92. 10.1002/path.5811 34599510

[B3] Chen R. Dou X. K. Dai M. S. Sun Y. Sun S. J. Wu Y. (2022). The role of dexmedetomidine in immune tissue and inflammatory diseases: A narrative review. Eur. Rev. Med. Pharmacol. Sci. 26, 8030–8038. 10.26355/eurrev_202211_30157 36394754

[B4] Dang S. Shen Y. Yin K. Zhang J. (2012). TREM-1 promotes pancreatitis-associated intestinal barrier dysfunction. Gastroenterol. Res. Pract. 2012, 720865. 10.1155/2012/720865 22611379 PMC3352574

[B5] Ferrero-Andrés A. Panisello-Roselló A. Roselló-Catafau J. Folch-Puy E. (2020). NLRP3 inflammasome-mediated inflammation in acute pancreatitis. Int. J. Mol. Sci. 21, 5386. 10.3390/ijms21155386 32751171 PMC7432368

[B6] Ge P. Luo Y. Okoye C. S. Chen H. Liu J. Zhang G. (2020). Intestinal barrier damage, systemic inflammatory response syndrome, and acute lung injury: A troublesome trio for acute pancreatitis. Biomed. Pharmacother. = Biomedecine Pharmacother. 132, 110770. 10.1016/j.biopha.2020.110770 33011613

[B7] Gui M. Zhao B. Huang J. Chen E. Qu H. Mao E. (2023). Pathogenesis and therapy of coagulation disorders in severe acute pancreatitis. J. Inflamm. Res. 16, 57–67. 10.2147/JIR.S388216 36636248 PMC9831125

[B8] He R. Wang Z. Dong S. Chen Z. Zhou W. (2022). Understanding necroptosis in pancreatic diseases. Biomolecules 12, 828. 10.3390/biom12060828 35740953 PMC9221205

[B9] Horibe M. Ravella B. Chandra S. Sharma A. Sato Y. Vege S. S. (2022). Trends in the incidence and etiology of acute pancreatitis from 2000 to 2016: A population-based study. Pancreatology 22, 828–829. 10.1016/j.pan.2022.07.002 35842376

[B32] Hu J. Kang H. Chen H. Yao J. Yi X. Tang W. (2020). Targeting neutrophil extracellular traps in severe acute pancreatitis treatment. Therap Adv Gastroenterol. 13, 1–20. 10.1177/1756284820974913 PMC7692350 33281940

[B10] Huang D. Y. Li Q. Shi C. Y. Hou C. Q. Miao Y. Shen H. B. (2020). Dexmedetomidine attenuates inflammation and pancreatic injury in a rat model of experimental severe acute pancreatitis via cholinergic anti-inflammatory pathway. Chin. Med. J. 133, 1073–1079. 10.1097/CM9.0000000000000766 32265428 PMC7213633

[B11] Jiang Y. Xia M. Huang Q. Ding D. Li Y. Zhang Z. (2019). Protective effect of dexmedetomidine against organ dysfunction in a two-hit model of hemorrhage/resuscitation and endotoxemia in rats. Braz J. Med. Biol. Res. 52, e7905. 10.1590/1414-431X20187905 30810621 PMC6393854

[B12] Kang H. Yang Y. Zhu L. Zhao X. Li J. Tang W. (2022). Role of neutrophil extracellular traps in inflammatory evolution in severe acute pancreatitis. Chin. Med. J. 135, 2773–2784. 10.1097/CM9.0000000000002359 36729096 PMC9945416

[B13] Leak L. Dixon S. J. (2023). Surveying the landscape of emerging and understudied cell death mechanisms. Biochimica biophysica acta. Mol. Cell Res. 1870, 119432. 10.1016/j.bbamcr.2023.119432 PMC9969746 36690038

[B14] Li H. Zhao L. Wang Y. Zhang M. C. Qiao C. (2022). Roles, detection, and visualization of neutrophil extracellular traps in acute pancreatitis. Front. Immunol. 13, 974821. 10.3389/fimmu.2022.974821 36032164 PMC9414080

[B15] Li Y. Pan Y. Gao L. Lu G. Zhang J. Xie X. (2018). Dexmedetomidine attenuates pancreatic injury and inflammatory response in mice with pancreatitis by possible reduction of NLRP3 activation and up-regulation of NET expression. Biochem. Biophys. Res. Commun. 495, 2439–2447. 10.1016/j.bbrc.2017.12.090 29269298

[B16] Liu D. Wen L. Wang Z. Hai Y. Yang D. Zhang Y. (2022). The mechanism of lung and intestinal injury in acute pancreatitis: A review. Front. Med. (Lausanne). 9, 904078. 10.3389/fmed.2022.904078 35872761 PMC9301017

[B17] Liu M. Wu W. Zhao Q. Feng Q. Wang W. (2015). High expression levels of trigger receptor expressed on myeloid cells-1 on neutrophils associated with increased severity of acute pancreatitis in mice. Biol. Pharm. Bull. 38, 1450–1457. 10.1248/bpb.b15-00057 26250893

[B18] Liu X. Li Y. Kang L. Wang Q. (2021). Recent advances in the clinical value and potential of dexmedetomidine. J. Inflamm. Res. 14, 7507–7527. 10.2147/JIR.S346089 35002284 PMC8724687

[B19] Liu Y. Yu Y. Zhang J. Wang C. (2019). The therapeutic effect of dexmedetomidine on protection from renal failure via inhibiting KDM5A in lipopolysaccharide-induced sepsis of mice. LIFE Sci. 239, 116868. 10.1016/j.lfs.2019.116868 31682847

[B20] Mercantepe F. Tumkaya L. Mercantepe T. Rakici S. Y. Ciftel S. Ciftel S. (2023). Radioprotective effects of α2-adrenergic receptor agonist dexmedetomidine on X-ray irradiation-induced pancreatic islet cell damage. Naunyn-Schmiedeberg's archives Pharmacol. 10.1007/s00210-023-02454-0 36877270

[B21] Moore J. Shehabi Y. Reade M. C. Bailey M. Fraser J. F. Murray L. (2022). Stress response during early sedation with dexmedetomidine compared with usual-care in ventilated critically ill patients. Crit. care (London, Engl. 26, 359. 10.1186/s13054-022-04237-0 PMC9682690 36419197

[B22] Nesvaderani M. Dhillon B. K. Chew T. Tang B. Baghela A. Hancock R. E. (2022). Gene expression profiling: Identification of novel pathways and potential biomarkers in severe acute pancreatitis. J. Am. Coll. Surg. 234, 803–815. 10.1097/XCS.0000000000000115 35426393

[B23] Schmidt J. Rattner D.W. Lewandrowski K. Compton C.C. Mandavilli U. Knoefel W.T. (1992). A better model of acute pancreatitis for evaluating therapy. Ann. Surg. 215, 44–56. 10.1097/00000658-199201000-00007 1731649 PMC1242369

[B24] Tovey Crutchfield E. C. Garnish S. E. Hildebrand J. M. (2021). The role of the key effector of necroptotic cell death, MLKL, in mouse models of disease. Biomolecules 11, 803. 10.3390/biom11060803 34071602 PMC8227991

[B25] Wang X. Zhang L. Li P. Zheng Y. Yang Y. Ji S. (2022). Apelin/APJ system in inflammation. Int. Immunopharmacol. 109, 108822. 10.1016/j.intimp.2022.108822 35605524

[B26] Wiegand A. Behal M. Robbins B. Bissell B. Pandya K. Mefford B. (2023). Niche roles for dexmedetomidine in the intensive care unit. Ann. Pharmacother. 31, 106002802211511. 10.1177/10600280221151170 36721323

[B27] Xia Z. N. Zong Y. Zhang Z. T. Chen J. K. Ma X. J. Liu Y. G. (2017). Dexmedetomidine protects against multi-organ dysfunction induced by heatstroke via sustaining the intestinal integrity. Shock 48, 260–269. 10.1097/SHK.0000000000000826 28709158

[B28] Xiang H. Guo F. Tao X. Zhou Q. Xia S. Deng D. (2021). Pancreatic ductal deletion of S100A9 alleviates acute pancreatitis by targeting VNN1-mediated ROS release to inhibit NLRP3 activation. Theranostics 11, 4467–4482. 10.7150/thno.54245 33754072 PMC7977474

[B29] Xiao M. Jiang C. F. Gao Q. Pan J. Zhang H. Wu S. N. (2023). Effect of dexmedetomidine on cardiac surgery patients. J. Cardiovasc. Pharmacol. 81, 104–113. 10.1097/FJC.0000000000001384 36607614

[B30] Yang Y. Zhang Y. Wen S. Cui Y. (2023). The optimal timing and intervention to reduce mortality for necrotizing pancreatitis: A systematic review and network meta-analysis. World J. Emerg. Surg. 18, 9. 10.1186/s13017-023-00479-7 36707836 PMC9883927

[B31] Zheng X. Zhao J. Wang S. Hu L. (2022). Research progress of antioxidant nanomaterials for acute pancreatitis. Molecules 27, 7238. 10.3390/molecules27217238 36364064 PMC9658789

